# A Mystery of Joint Pain: Is It Rheumatoid Arthritis (RA) or Hereditary Hemochromatosis (HH)?

**DOI:** 10.7759/cureus.33037

**Published:** 2022-12-28

**Authors:** Hlaing Myat Chit Su, Kiran Putchakayala

**Affiliations:** 1 Rheumatology/General Medicine, Whiston Hospital, Whiston, GBR; 2 Rheumatology, Leighton Hospital, Leighton, GBR

**Keywords:** genetic etiology, and misdiagnosis, chronic joint pain, hereditary haemochromatosis, : rheumatoid arthritis

## Abstract

A 58-year-old lady with a previous diagnosis of rheumatoid arthritis (RA) was referred to Rheumatology to manage her joint pains. On evaluation, it was noted that the lady did not have any signs of synovial inflammation. The patient had a negative anti-cyclic citrullinated peptide (anti-CCP) (<0.5) and negative rheumatoid factor (RF) (<10) together with high ferritin (1,507 µg/L) which led to consideration of hereditary hemochromatosis (HH) rather than RA. She was then referred to Hematology for regular venesection which settled her symptoms. This case report highlights the importance of considering HH as a differential diagnosis in patients with chronic arthritis particularly if there are no clinical signs and negative tests for RA. More retrospective studies will be needed to quantify how many cases of hemochromatosis arthropathy have been mistakenly diagnosed as RA.

## Introduction

Hereditary hemochromatosis (HH) is an autosomal recessive genetic disorder due to mutations of the homeostatic iron regulator (HFE) gene (located on the short arm of chromosome 6). It is characterized by increased intestinal iron absorption causing iron overload in internal organs [1]. It can manifest in multiple organs including joints causing arthropathy mimicking features of rheumatoid arthritis (RA). In approximately half of the cases of HH, chronic progressive arthritis affecting metacarpophalangeal (MCP) joints and proximal interphalangeal (PIP) joints and wrists is the presenting complaint and can sometimes perplex physicians and lead to a diagnosis of inflammatory arthritis [2].

## Case presentation

A 58-year-old lady was referred to the Rheumatology outpatient department by her General Practitioner (GP) due to increasing pain in the neck and low back pain. She was previously diagnosed as possible RA over 10 years ago in another hospital where she was initially treated with methotrexate (MTX) which was later stopped due to side effects. We do not have any documentation regarding why and how she was initially diagnosed with RA. She denied any family history of psoriasis or inflammatory bowel disease.

**Figure 1:**
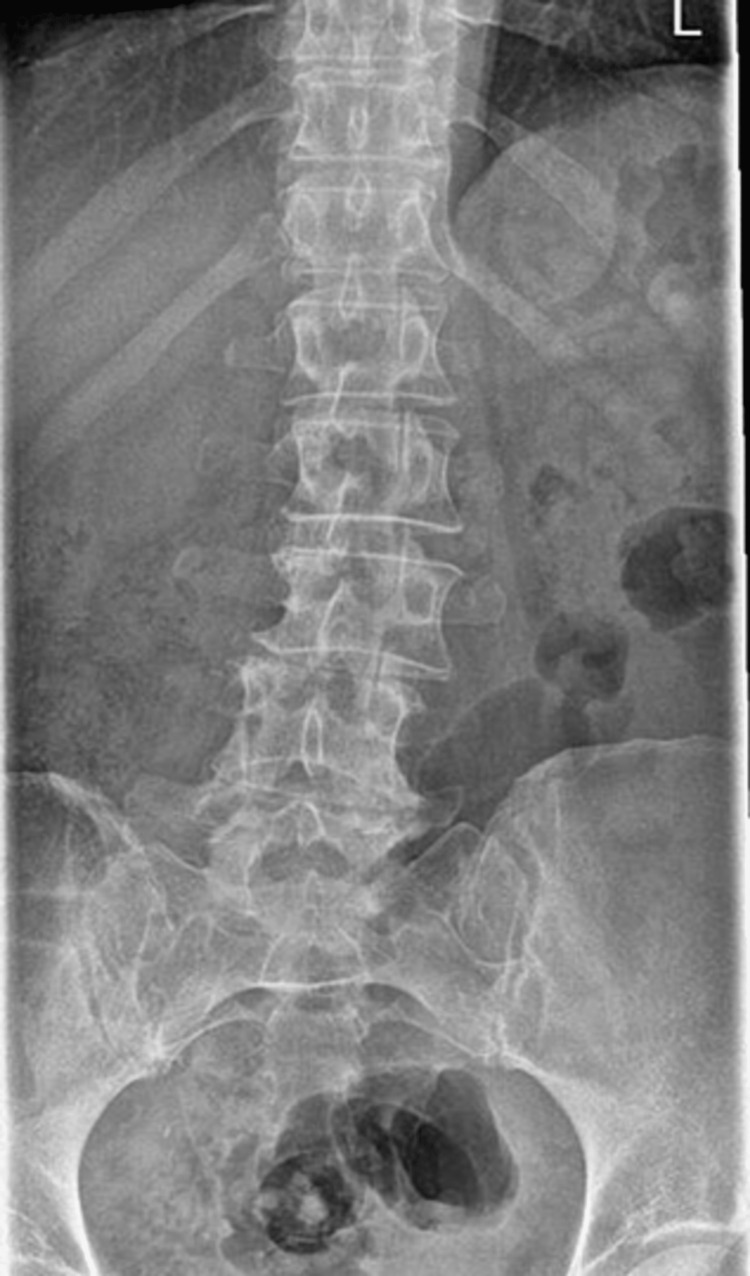
XR of the lumbar spine

**Figure 2:**
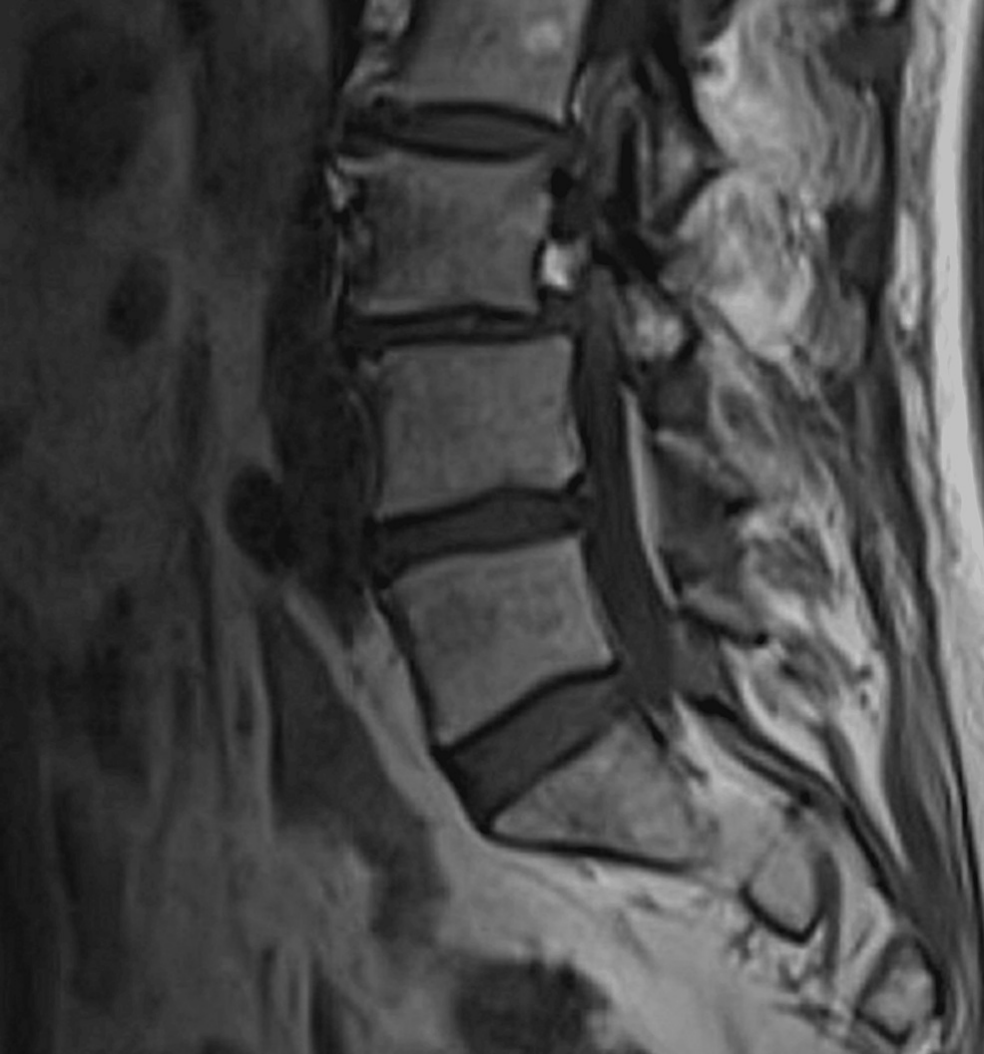
MRI lumbar spine

**Figure 3:**
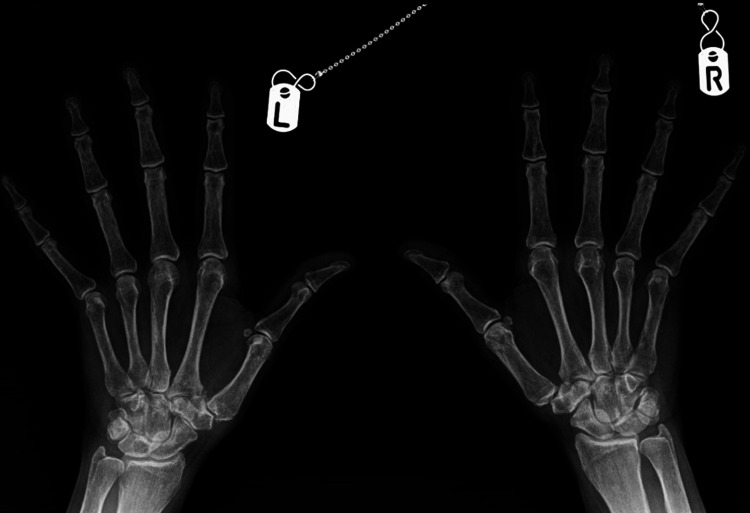
XR hands of the patient

**Figure 4:**
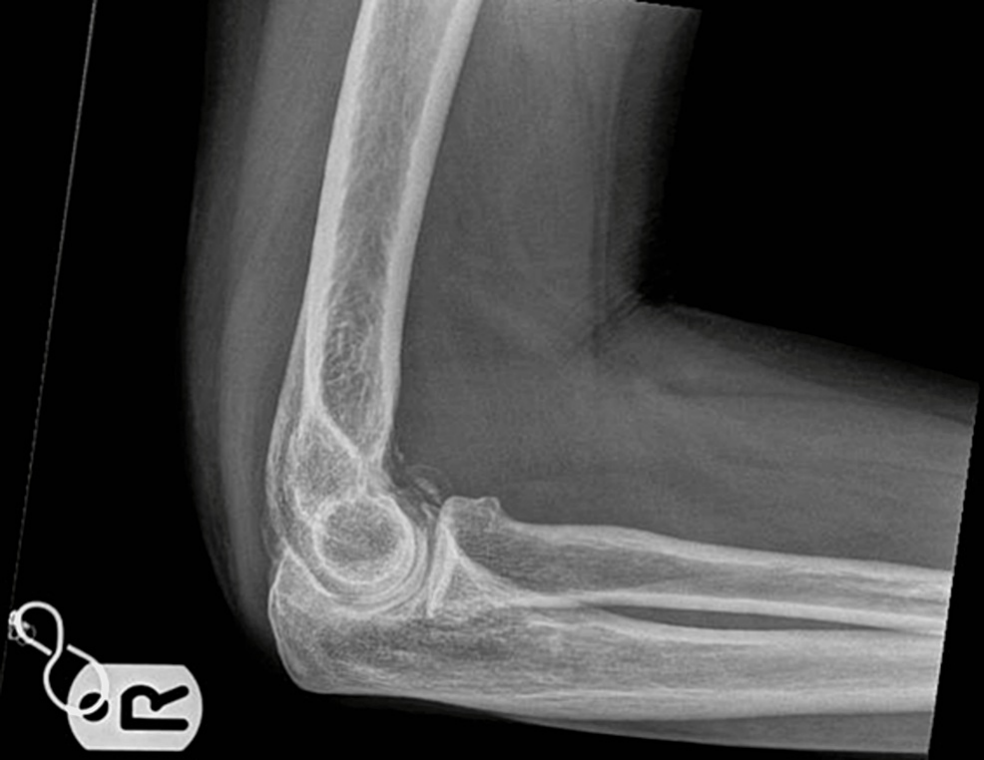
XR of the right elbow

**Figure 5:**
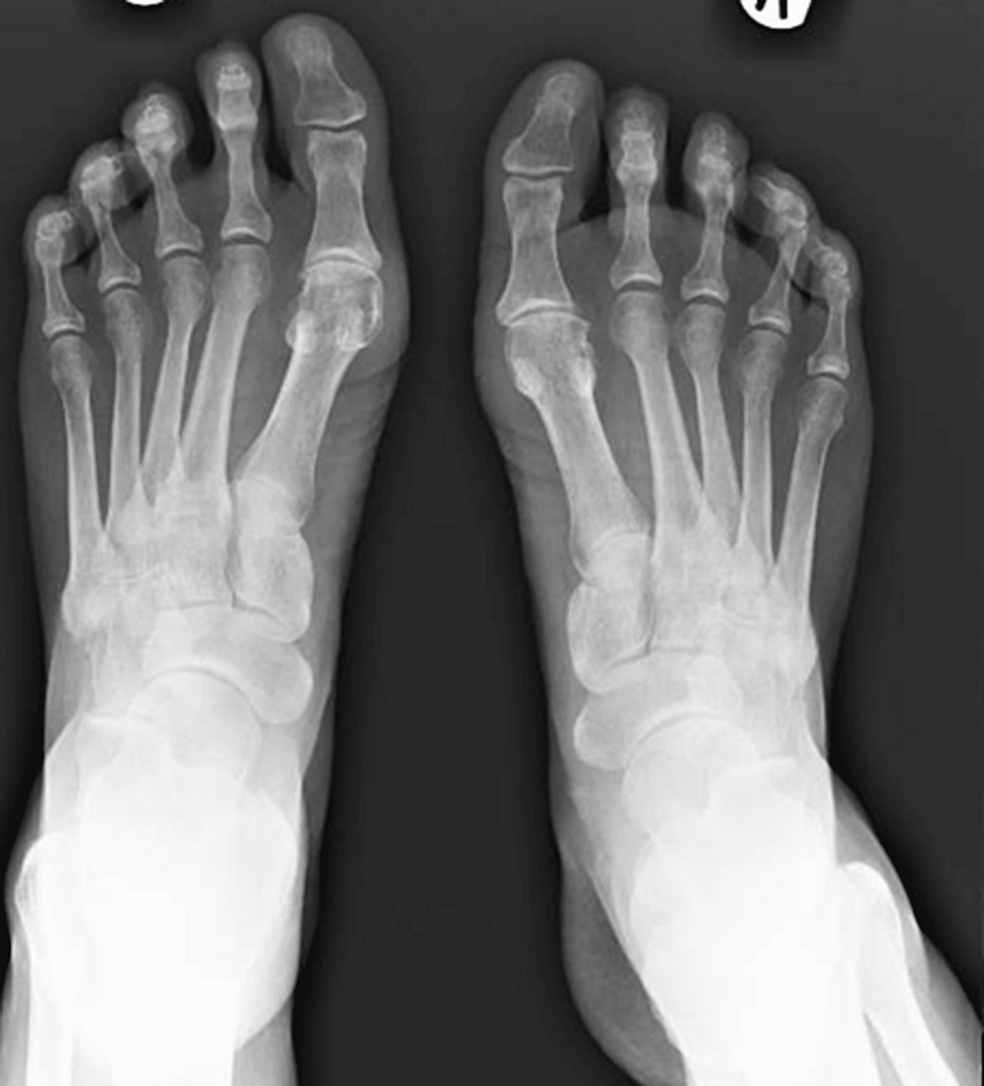
XR of the feet

On clinical examination, her lumbar spine movement was restricted significantly. No active synovitis was noted on MCP joints or interphalangeal joints of the hands or wrists. Figure 1 shows an x-ray of her lumbar spine which showed mild scoliosis of the lumbar spine convex to the left with the anomalous articulation of L5 and S1 on the right, marked intervertebral space narrowing at L3-L4 and moderate disc space narrowing at L4-L5. MRI lumbar spine in figure 2 showed no evidence of recent or historic vertebral fracturing but indicated L3/L4 and L4/L5 spondylotic changes with central high-grade stenosis with transiting neural impingement. Figures 3-5 showed no abnormalities in hands, elbows, and feet respectively.

The plan was made to revisit the previous diagnosis of inflammatory arthritis/RA as there were no active inflammatory arthritis features. The other differentials would be seronegative arthritis or any systemic causes that could mimic RA such as polyarticular gout, or HH.

On biochemical investigations as shown in Table 1, her full blood count was normal with an erythrocyte sedimentation rate (ESR) of 2 milimeters per hour (mL/hr), and C-reactive protein (CRP) 5 milligrams per liter (mg/L). her human leucocyte antibody B (HLA-B) 27, anti-nuclear Ab (ANA), anti-centromere Ab, anti-cyclic citrullinated peptide (anti-CCP) antibody, and rheumatoid factor (RF) were negative. The iron profile showed that Ferritin 1507 micrograms per liter (mcg/L), Iron 35 micromoles per liter (mcmol/L), transferrin 1.5 grams per liter (g/L), and transferrin saturation 93 percent (%). A hemochromatosis gene screen revealed that she is homozygous for the C282Y mutation resulting in a tyrosine for cysteine substitution at amino acid 282. She was referred to Hematology for weekly venesections. Her GP was advised to screen her siblings and her husband in view of her concerns about her children being affected.

**Table 1:** Biochemical investigations of the patient

Tests	Results	units	Normal range
Haemoglobin	140	Grams per liter (g/L)	115-165
White cell count	5.5	10^9 ^per liters	4-11
Platelet	192	10^9 ^per liters	150-450
C-reactive protein	5	Miligrams per liter (mg/L)	0-7
Erythrocyte sedimentation rate	2	Milimeters per hour (ml/hr)	2-16
Anit-cyclic citrullinated peptide Antibody	<0.5	kilo International units per liter(kIU/L)	0-2.9
Rheumatoid Factor	<10	Kilo International units per liter (kIU/L)	0-14
Human leucocyte antigen B27	Negative		
Anti-nuclear Antibody	Negative		
Anti-centromere Antibody	<0.2	AI	0-0.9
Ferritin	1507	Micrograms per liter (mcg/L)	10-150
Iron	35	Micromoles per liter (mcmol/L)	9-30
Transferrin	1.5	Grams per liter (g/L)	2-3.6
Transferrin saturation	93	Percentage (%)	15-45

**Figure 6:**
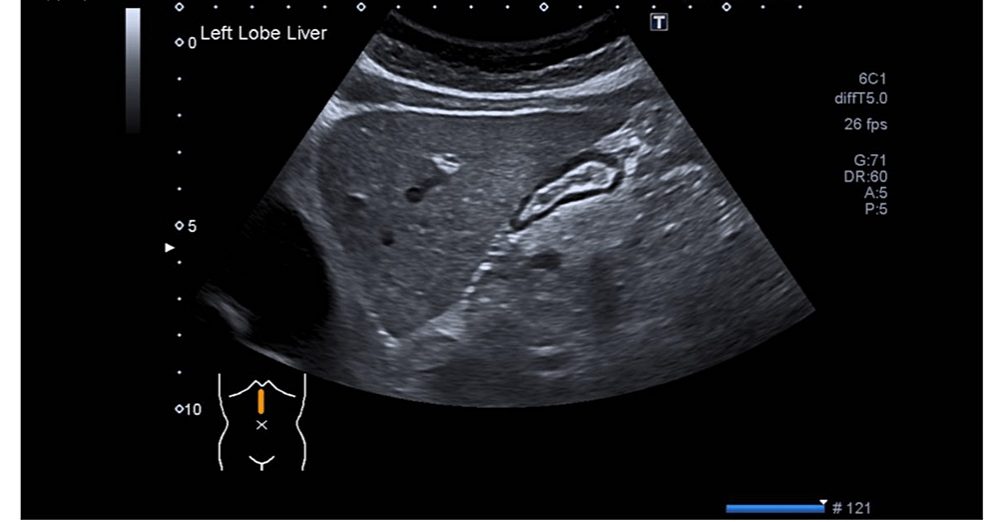
US abdomen

During the follow-up, she had been feeling much better. She had also been seen by gastroenterologists who arranged a further non-invasive liver screen with the US scan of the abdomen as shown in Figure 6. The liver appears mildly, and uniformly increased in echogenicity consistent with fatty infiltration. Liver elastography showed an increased acoustic radiation force impulse (ARFI) reading of 1.88 meters per second(m/s) which indicates F2 fibrosis. Non-invasive liver screening blood showed negative for gastric parietal cell antibody, liver kidney microsomal antibody, anti-mitochondrial antibody, and smooth muscle antibody. On genetic screening, her siblings and three children (aged 25, 24, and 22) are noted to be carriers of the hemostatic iron regulator (HFE) gene.

## Discussion

HH, an autosomal recessive genetic condition, is the most common cause of primary iron overload, associated with C282Y alleles [3]. Clinical presentations of HH range from bronze skin to diabetes, cirrhosis, and hepatocellular carcinoma [4]. However, it is interesting to note that arthropathy is the most common presenting complaint in patients with HH predating many years before the diagnosis of HH [4]. Therefore, it is not surprising that it is often confused with other rheumatic diseases including Rheumatoid Arthritis (RA). In a similar case presented by Ryan Hum and Pauline Ho [5], interestingly, US hands and feet showed active evidence of synovitis explaining the association of possible chondrocalcinosis associated with HH [5]. HH arthropathy can present as typical hand osteoarthritis (HOA) presentation. However, the latter would more severely affect the MCP joints thereby affecting hand functions more [6].

Physicians should have a high index of suspicion when a patient with presumed inflammatory arthritis is not responding to treatment or does not have any signs of active synovitis on complete physical examination. Hemochromatosis should be suspected when there is a high iron profile and positive for HFE genotyping while negative for RF and anti-CCP [7]. It is, however, possible to find normal serum iron and ferritin level with high transferrin saturation levels in HH according to Akgol et al. [7]. Feder et al. have determined that transferrin saturation level is the most sensitive analysis of iron overload in the body and that the cut-off value is over 45% [8].

Misdiagnosis as RA like in this case potentially delayed the treatment and exposed patients to steroid burden and side effects of disease-modifying agents such as MTX [9]. The symptoms were also not responsive to the disease-modifying treatment regime possibly leading to the escalation of treatment to biological therapy.

Moreover, it is essential for patients with HH to have genetic testing of family members to predict the likelihood of disease incidence in future generations [3]. Misdiagnosis can, therefore, not only adversely affect the patient but also could potentially cause the immediate family to suffer in the future.

As HH has low penetrance, it is less likely to be cost-effective to do genetic screening in normal populations. However, ferritin screening in patients with chronic progressive arthritis should be beneficial as the bulk cost of ferritin testing is $5 per test [10].

## Conclusions

It is important for physicians to know that HH is an important differential diagnosis of RA. If misdiagnosed with RA as in this case, the symptoms would not have been relieved, and also there would be treatment burdens such as steroids and DMARDs including MTX. Moreover, it can affect the future generation of the patient as HH is a serious genetic disorder. It is thus essential to screen hemochromatosis on a new diagnosis of suspected RA patients with iron studies. More studies are recommended to highlight the importance of missed diagnoses and increase awareness of primary care and general internal medicine physicians.
